# Sterile Insect Technique (SIT) field trial targeting the suppression of *Aedes albopictus* in Greece[Fn FN1]

**DOI:** 10.1051/parasite/2024020

**Published:** 2024-03-26

**Authors:** Georgios Balatsos, Vasileios Karras, Arianna Puggioli, Fabrizio Balestrino, Romeo Bellini, Dimitrios P. Papachristos, Panagiotis G. Milonas, Nikos T. Papadopoulos, Marco Malfacini, Marco Carrieri, Apostolos Kapranas, Wadaka Mamai, George Mastronikolos, Ioanna Lytra, Jérémy Bouyer, Antonios Michaelakis

**Affiliations:** 1 Scientific Directorate of Entomology and Agricultural Zoology, Benaki Phytopathological Institute 14561 Kifissia Greece; 2 Centro Agricoltura Ambiente “G. Nicoli” 40014 Crevalcore Italy; 3 Insect Pest Control Laboratory, Joint FAO/IAEA Programme of Nuclear Techniques in Food and Agriculture Seibersdorf A-2444 Vienna Austria; 4 Department of Agriculture, Crop Production and Rural Environment, University of Thessaly 38446 Magnisias Greece; 5 Laboratory of Applied Zoology and Parasitology (Entomology), School of Agriculture, Aristotle University of Thessaloniki 54124 Thessaloniki Greece; 6 ASTRE, CIRAD 34398 Montpellier France; 7 ASTRE, Cirad, INRAE, Univ. Montpellier, Plateforme Technologique CYROI Sainte-Clotilde La Réunion France

**Keywords:** Egg density, Egg hatching rates, Sterile male insect transportation, Mosquito borne diseases, Public health

## Abstract

The sterile insect technique (SIT) involves releasing large numbers of sterile males to outcompete wild males in mating with females, leading to a decline in pest populations. In the current study, we conducted a suppression trial in Greece against the invasive dengue vector mosquito *Aedes albopictus* (Skuse) through the weekly release of sterile males for 22 weeks from June to September 2019. Our approach included the long-distance transport of sterile mosquitoes, and their release at a density of 2,547 ± 159 sterile males per hectare per week as part of an area-wide integrated pest management strategy (AW-IPM). The repeated releases of sterile males resulted in a gradual reduction in egg density, reaching 78% from mid-June to early September. This reduction remained between 70% and 78% for four weeks after the end of the releases. Additionally, in the SIT intervention area, the ovitrap index, representing the percentage of traps containing eggs, remained lower throughout the trial than in the control area. This trial represents a significant advance in the field of mosquito control, as it explores the viability and efficacy of producing and transporting sterile males from a distant facility to the release area. Our results provide valuable insights for future SIT programmes targeting *Ae. Albopictus*, and the methodology we employed can serve as a starting point for developing more refined and effective release protocols, including the transportation of sterile males over long distances from production units to intervention areas.

## Abstract (fr)

La technique de l’insecte stérile (TIS) consiste à libérer un grand nombre de mâles stériles pour supplanter les mâles sauvages lors de l’accouplement avec les femelles, entraînant ainsi un déclin des populations de nuisibles. Dans la présente étude, nous avons mené un essai de suppression en Grèce contre le moustique vecteur invasif de la dengue, *Aedes albopictus* (Skuse), par le biais de la libération hebdomadaire de mâles stériles pendant 22 semaines de juin à septembre 2019. Notre approche comprenait le transport sur de longues distances de moustiques stériles, et leur lâcher à une densité de 2 547 ± 159 mâles stériles par hectare et par semaine dans le cadre d’une stratégie de lutte intégrée contre les nuisibles à l’échelle de la zone (AW-IPM). Les lâchers répétés de mâles stériles ont entraîné une réduction progressive de la densité des œufs, atteignant 78 % de la mi-juin au début septembre. Cette réduction est restée entre 70 % et 78 % pendant quatre semaines après la fin des lâchers. De plus, dans la zone d’intervention de la TIS, l’indice d’oviposition, représentant le pourcentage de pièges contenant des œufs, est resté plus faible que dans la zone témoin tout au long de l’essai. Cet essai représente une avancée significative dans le domaine de la lutte contre les moustiques, car il explore la viabilité et l’efficacité de la production et du transport de mâles stériles depuis une installation éloignée vers la zone de lâcher. Nos résultats fournissent des informations précieuses pour les futurs programmes de TIS ciblant *Ae. albopictus* et la méthodologie que nous avons utilisée pourra servir de point de départ pour développer des protocoles de libération plus raffinés et plus efficaces, y compris le transport de mâles stériles sur de longues distances depuis les unités de production jusqu’aux zones d’intervention.

## Introduction

*Aedes* invasive mosquito species (AIM) have been recorded in many European and Mediterranean countries since the first detection of *Aedes albopictus* (Skuse), in the 1970s in Albania [10]. AIM mosquito species can enter and establish permanent populations in new areas with favourable environmental and climatic conditions. This phenomenon is being further exacerbated by climate change [16]. Consequently, new public health risks are faced, including the increasing incidence of mosquito-borne diseases (MBDs) such as chikungunya and dengue, which are currently emerging in different European countries [19, 20].

The Asian tiger mosquito (*Ae. albopictus*) is the main AIM species in Europe, already causing public health problems as well as intense nuisance [10]. Due to its aggressive and day biting behaviour, it is affecting public perception regarding the effectiveness of mosquito control programmes. The establishment of *Ae. albopictus* in urban and semi-urban areas challenges the existing mosquito control programmes that are coordinated by local authorities and executed mainly by various private entities [1].

The management plan to control *Ae. albopictus* is thus a complex system that includes coordinated actions to control its populations. Conventional control programmes applied successfully against marshland mosquitoes are less effective in controlling the population of *Ae. albopictus* in urban areas due to its unique bioecology, *e.g.*, cryptic breeding behaviour, daily biting behaviour and the dispersion of breeding sites [10, 26]. Cases of resistance to insecticides in *Ae. albopictus* populations in certain European countries has been reported [32], whereas there are limited options available for larvicidal use. Therefore, there is a need to implement alternatives to chemically based vector control methods and strategies, such as the sterile insect technique (SIT) and citizen science [24, 31].

The SIT method is based on the release of large numbers of sterile males to outcompete wild males for mating with females. The SIT involves three key steps: production of target species in large numbers under controlled environments; male sterilisation using ionising radiation and systematic release of sterile individuals within the target area [25]. This method relies on the sterile males’ ability to mate with wild females, thereby decreasing the overall reproductive success of the population. Its successful application has led to improvements in pest control, minimising the need for chemical interventions and reducing associated environmental risks [18]. This is also in accordance with the EU policy as set out in EU Regulation 528/2012 aiming to reduce the use of biocides.

The efficacy of SIT can be enhanced through further improvements, and it can be effectively integrated with other vector control strategies, including source reduction [31]. Source reduction refers to the elimination of breeding habitats, aiming to minimise the availability of suitable sites for mosquito reproduction. Despite the extensive success of the SIT in controlling agricultural insect pests, its implementation for mosquito vectors of human diseases is currently at an early stage of development. Pilot trials are being conducted to assess the viability and efficacy of SIT as a method for controlling mosquito populations, specifically targeting species belonging to the *Aedes* spp.

In 2019, a suppression trial took place in Vravrona located in the Attica Region of Greece. Prior to this trial, in the 2018 summer period, a door-to-door strategy had previously been implemented, raising public awareness through knowledge, attitude, and practice (KAP) surveys aiming to reduce breeding sites both in private and public areas [34]. Additionally, a pilot release of sterile males had been conducted at the end of the previous mosquito season in 2018 [2]. The suppression trial was conducted throughout the mosquito season and the primary goal was to evaluate the efficacy of the SIT in suppressing *Ae. albopictus* populations in Greece, within the context of an area wide (AW) approach. Additionally, the study aimed to explore the long-distance transfer of sterile male mosquitoes. Therefore, all sterile males were exclusively produced at the Centro Agricoltura Ambiente “G. Nicoli” (CAA, Italy) facility and transported to the specified release plot [2, 29]. To our knowledge, this study is the first attempt to use the SIT for mosquito suppression, which is based on long distance production of sterile males.

## Materials and methods

### Description of plots

**Figure 1:**
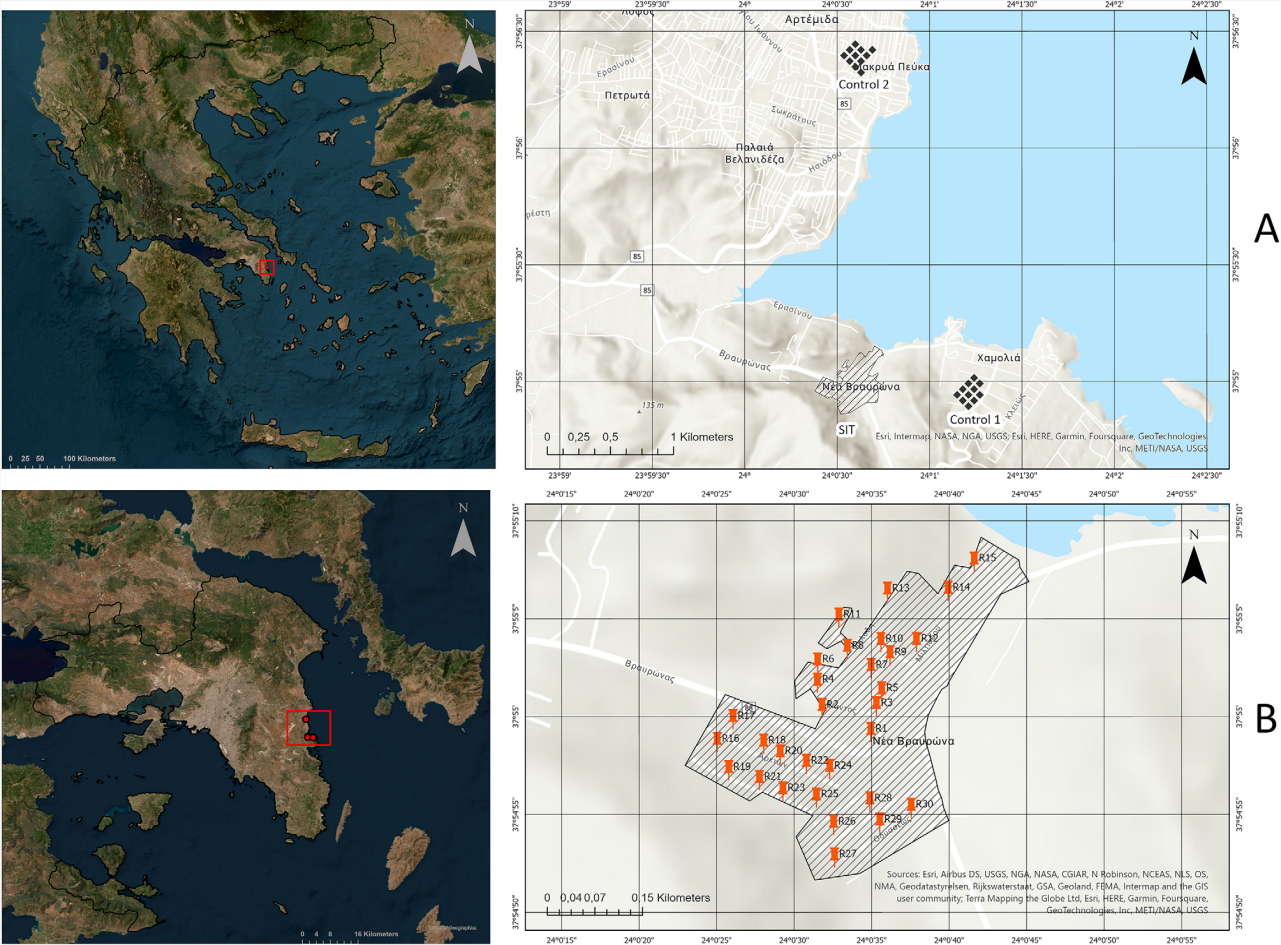
Geographic position of the SIT plots and the respective control plots C1 and C2 (A) and distribution of releasing points within the SIT plot (B).

The suppression trial took place in Vravrona, Markopoulo municipality, Attica Region (East Regional Unit) of Greece located east of Athens (SIT plot) (Fig. 1 and Table 1). The total focal area of the Vravrona site was almost 10 ha. To the north, the area is surrounded by the sea, while to the south, west, and east, the nearest urban areas are approximately 1.5 km away. Therefore, it was isolated from other urban areas since the focal area was surrounded by high hills covered by montane forests. Based on the previous trial, there was no significant difference between the control and SIT plot [2]. To compare the efficacy of SIT in the current study with that of the previous study, we established two control plots (5 ha each) in semi-urban areas close to the release plot. Control 1 is located 1 km east of the SIT plot, and control 2, more than 4 km north of the SIT area (Fig. 1 and Table 1). Moreover, in collaboration with the Municipality of Markopoulo, no chemical treatments were implemented during the trial in public areas falling under the municipality’s authority.

**Table 1:** Description of the study plot and the respective control plots including the number of ovitraps deployed to assess the efficacy of the SIT trial.

Plot	Activity	Description	Surface area (ha)	No. Ovitraps
SIT	Sterile male releases	An isolated semi-urban area in Vravrona (Municipality of Markopoulo, Attika Region)	10	30
Control 1	No intervention	Semi-urban area 1.5 km east from the SIT plot (Municipality of Markopoulo, Attika Region)	5	15
Control 2	No intervention	Semi-urban area 4.5 km north from the SIT plot (Municipality Spata-Artemida, Attika Region)	5	15

### Sterile male production and handling

All sterile *Ae. albopictus* males (Greek strain) were produced at the CAA mass rearing facility using methods described in Bellini *et al*. [8] and Malfacini *et al*. [28]. The mosquito colony used for the current suppression trial originated from eggs collected in Vravrona in 2017, 2018 and 2019 [2]. Mosquito larvae were mass reared according to the IAEA protocols [3, 27]. Males were irradiated at the pupal stage with a dose of 35 Gy using an IBL 437 irradiator (CIS Bio International, Saclay, France) equipped with a Cs-137 linear source. Based on this protocol, this irradiation level strikes the optimal balance between sterility and the performance of sterile males. Furthermore, the age of the male mosquito pupa at the time of irradiation (calculated from pupation to the irradiation moment) exerts a substantial influence on both the achieved sterility level and the quality of the resulting adults [4].

### Transportation of sterile males and release

Male mosquitoes were chilled in a large cooling cabinet (8 ± 1 °C, 85 ± 5% RH) for approximately one hour before packaging. The cold-shock anaesthetised sterile males were then transferred to small plastic cylindrical containers (plastic box 5 cm diameter, 5 cm height, 80 cc capacity) placed and sealed with tape inside a larger plastic container (PP plastic, 20 × 15 × 6 h cm, 1,800 cc capacity). Up to three of these plastic containers were stacked vertically and packed inside a polystyrene container with adequate quantity of phase-changing materials (PCM) to maintain a temperature around 12 °C and delivered by express courier service from the production facility (Centro Agricoltura Ambiente “G. Nicoli” – CAA) to Athens as described in Mastronikolos [29]. Upon arrival, sterile males were immediately transferred to the release plot.

Cup shaped paper containers (paper box, diameter of upper base 12 cm and lower base 11 cm, 8 cm height) were used to release sterile males. Each paper container (release cup) was previously hardened on its inner surface to allow the sterile male mosquitoes to rest. Each container was hung by rope on one of the 30 predefined permanent release points (*e.g.*, tree branches, fences, *etc*.) (Fig. 1). Sterile males were released on a weekly basis for 22 weeks (from 03 May to 04 October 2019).

All releases were conducted approximately 1.5 h after receipt of shipments from Italy, in the 30 predefined permanent stations (release points) established in the SIT plot. Before releasing them, the anesthetised sterile males were transferred into the release cup, stoppered with mesh, which contained a 10% sugar solution, to regain their activity. After one hour, all paper containers (release cups) were distributed to the release sites. Some vaseline (Vaseline Original Pure Petroleum Jelly, Unilever, London, UK) was applied on the hanging rope to prevent possible access of predatory insects (e.g., ants). The release time lasted ca. 30 min (Video 1 available in Supplementary material). Sterile males that died during the release process or were unable to fly from the paper containers were collected and counted.

### Entomological monitoring to assess the impact of SIT application

To assess the impact of sterile male releases on the population dynamics of *Ae. albopictus*, we established a network of ovitraps, each consisting of a 1.5 L black plastic container accompanied by a wooden strip measuring 150 × 18 × 1.6 mm. In all plots, including both the SIT and control, we deployed three ovitraps per hectare, totalling 60 ovitraps (Table 1). We complied with the standard operational procedures for ovitrap field management, as outlined in Annexes 1 and 2 of Bellini *et al.* [10]. Throughout the year 2019, all ovitraps underwent weekly inspections, starting from 01 January and concluding on 31 December. Table 2 provides a comprehensive summary of weekly releases, including the quantity of mosquitoes delivered per week, the associated mortality rate, and the estimated density of sterile males released per hectare.

**Table 2:** Descriptive data of the 22 weekly releases of sterile males carried out in Greece in 2019.

Parameters	Mean ± SD	Total season
No. sterile males delivered (sent from Italy)	30,091 ± 8,065	662,000
Residual presence of females (%)	0.76 ± 0.12	–
Duration of transportation (h)	21.77 ± 0.58	–
Mean temperature during transportation (°C)	12.08 ± 1.90	–
Mortality of sterile males during transport (%)	16.19 ± 1.62	–
No. sterile males/release/ha (after mortality)	2,547 ± 159.3	585,810

### Weather and environmental parameters

**Figure 2:**
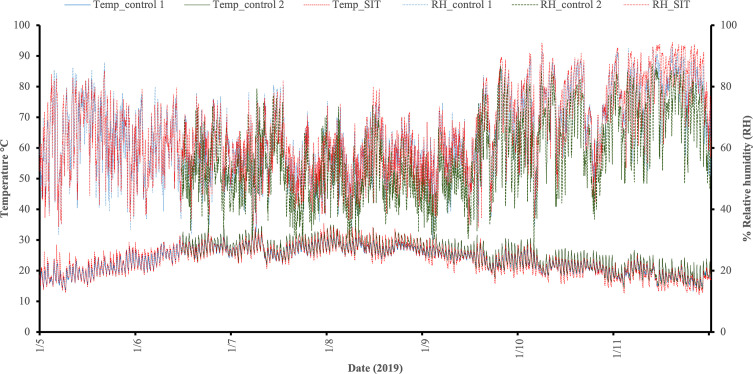
Temperature and Relative Humidity (RH) data at the SIT and control plots [SIT: 65.030 ± 0.185 RH and 23.414 ± 0.062 °C], [Control 1 (C1): 63.796 ± 0.178 RH and 23.315 ± 0.056 °C], [Control 2 (C2): 57.887 ± 0.205 RH and 25.175 ± 0.067 °C].

In each plot (SIT and controls), the temperature and RH was recorded by a temperature-humidity logger with an internal sensor (type Ebro EBI 20 TH1, Xylem Analytics Germany Sales GmbH & Co, Weilheim, Germany). These records are provided in Figure 2.

### Assessment of egg hatching rates

The oviposition substrates were collected weekly and managed carefully to protect eggs from desiccation during transportation to the laboratory (Annexes 1 and 2 in Bellini *et al*. [10]). At the laboratory, the oviposition substrates were left at 25 ± 1 °C, 80% RH, 14:10 L:D for one day and then placed in a container with a saturated solution of potassium sulphate (K_2_SO_4_) to complete embryonation for a minimum of six days. A protocol described in Bellini *et al*. [8, 9] was adopted for egg hatching. The steps involved in egg collection and storage of the oviposition substrate are crucial in assessing the hatching of eggs. In Table 3, we describe each step, including important information and notes that should be taken into account. This comprehensive summary aims to assist in achieving successful egg hatching outcomes.

**Table 3:** Description of steps to be followed to assess egg hatch.

Egg collection	**Step 1***.* Egg collection (oviposition substrates) from ovitraps and preserving them wet (use wet kitchen paper to wrap them).
	*Important note*: Avoid egg dryness during the trip from the field to the laboratory (avoid leaving the eggs in a car, in the sun).
Storage	**Step 2***.* Oviposition substrates should be left in the wet paper under standard laboratory conditions (25 ± 1 °C, 80% RH, 14:10 L) for 1 day to dry.
	**Step 3**. The next day, the oviposition substrates are placed in a sealed plastic container with vapours of potassium sulphate (K_2_SO_4_) under standard laboratory conditions. In each plastic container, 100 mL of a saturated solution (potassium sulphate: 120 g/L) was used. The oviposition substrates are left to embryonate under K_2_SO_4_ vapours for one week.
Hatching solution	**Step 4***.* Preparation of hatching solution. The maternal solution is prepared using 12.5 g broth (Nutrient Broth OXOID) + 2.5 g yeast powder/100 mL of deionised water.
	*Important note*: The hatching solution must be used immediately and cannot be stored.
Hatching	**Step 5a***.* 1st hatching. For egg hatching, 1 L volume glass jars with caps are used. 700 mL of deionised water and 2 mL of the hatching solution are added in each glass jar. Five oviposition substrates are put in one glass jar. The jars must be hermetically closed and opened 20–24 h later (not before 16 h). Then, hatched and unwatched eggs are counted. For the evaluation of the hatching, it is recommended to examine the eggs under the microscope rather than counting larvae. Sterile eggs are collapsed or exploded.
	**Step 5b**. If the hatching rate in control is less than 80%, a second hatching assessment must be applied for the substrates collected both in control and in SIT areas. After Step 5a, the eggs are left to dry for 3–4 days and then repeat the hatching protocol.

### Statistical analyses

The induced sterility (S) and decreased egg density (D) were calculated using the equations:

$$\begin{array}{c} S=(\mathrm{PW}-\mathrm{PS})/\mathrm{PW}\\ D=(\mathrm{ES}-\mathrm{EW})/\mathrm{EW} \end{array}$$

where ES and EW are the mean number of eggs per ovitrap per week in SIT and control plot, while PS and PW are the percentage of hatched eggs, in the SIT and in the control plot, respectively [7].

Generalised Poisson linear mixed models (GLMM) were used to assess the effect of SIT on egg density and binomial generalised linear mixed models were used to test the effect of treatment on egg sterility (hatch rate). Treatment, duration of treatment (weeks) and their first order interaction were used as fixed effects and trap numbers and dates were used as random effects. The full models were compared to simpler models using the second-order Akaike Information Criterion [15, 23]. The analysis was conducted until 25 October 2019 after which the oviposition activity and hatch rates dropped in all plots due to diapause.

## Results

### Transportation of sterile males and release

Mortality of sterile males during transport from Italy to the release point in Greece was on average 16.19 ± 1.62%. The duration of transportation from the CAA mass rearing facility in Italy to the field release plots in Vravrona was approximately 21 h (Table 2).

### Release of sterile males

For 22 weeks, 03 May to 04 October (2019), 662,000 sterile males were released. The mean release density per hectare per week was 2,547 (±159.3) sterile males, with an average female contamination of 0.76 ± 0.12% (Table 2).

### Assessment of egg hatching rate

**Figure 3:**
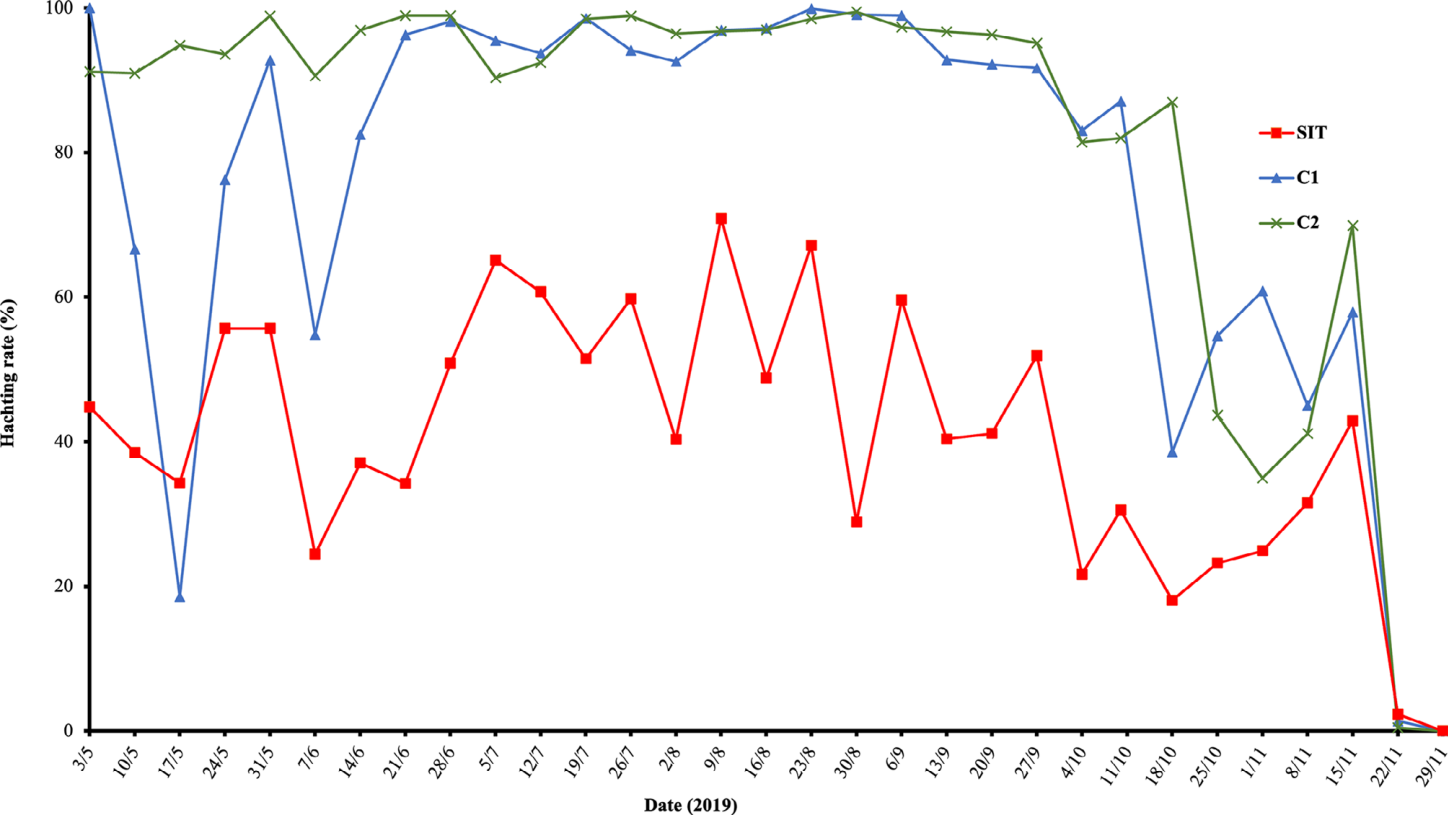
Hatching rates at SIT and control plots. The hatching rates of collected eggs (oviposition substrates) from 60 ovitraps were assessed weekly.

**Figure 4:**
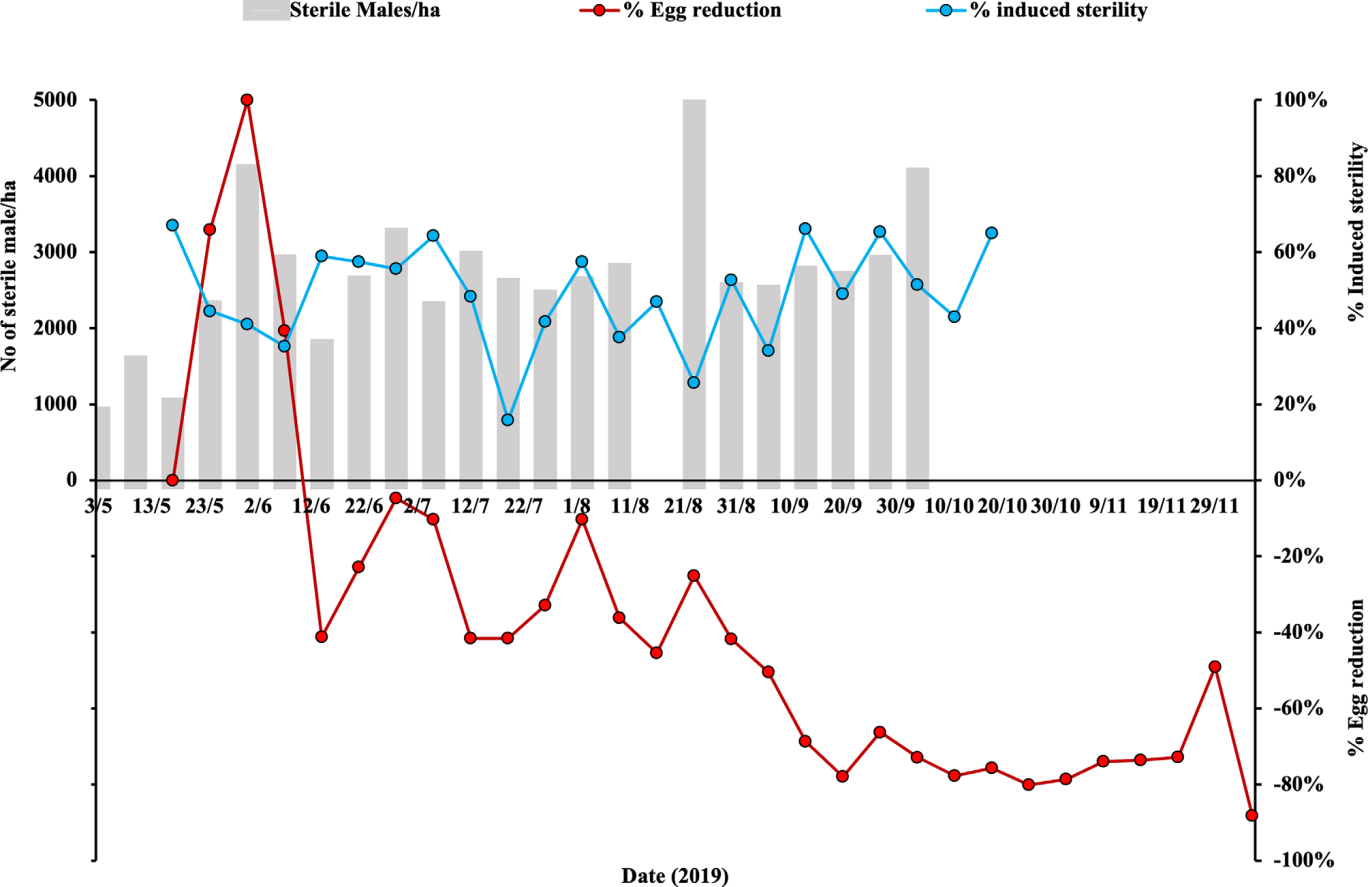
Seasonal patterns of induced sterility and egg reduction at the SIT plot as compared with the control area (right *Y* axis). The grey bars indicate the number of sterile males released per hectare (ha) per week from 03 May to 04 October (2019) at the SIT plot (left *Y* axis).

Egg hatching rates were higher overall in control 2 plot than in control 1 and SIT plots (*p* < 10^−3^, Table 4). Hatch rates were constant over time in control 2 (*p* = 0.50), increased in control 1 (*p* < 0.001) and decreased in the SIT plot (*p* < 0.01) (Fig. 3). The seasonal average of the rate of induced sterility (S) from 03 May to 04 October 2019 was 48.87% (±13.57) with a minimum of 15.80% during the period 05–12 July 2019 (high temperatures; 34 °C) and a maximum of 77.95% from 30 August to 5 September (Fig. 4).

**Table 4:** Fixed-effects coefficients of a mixed-effect binomial model of the impact of treatment, time and their interaction on the hatch rate of eggs of *Aedes albopictus* (1,426 observations, 60 traps, 24 collection dates, Control 2 site used as a reference).

Fixed effects	Value	Std. error	*z*-value	*p*-value
Intercept	3.28166	0.35249	9.310	<2e−16
Control 1	−2.07499	0.21090	−9.839	<2e−16
SIT	−3.23211	0.18347	−17.617	<2e−16
Time	−0.06215	0.09294	−0.669	0.50365
Control 1: Time	0.42256	0.02420	17.459	<2e−16
SIT: Time	−0.06015	0.02196	−2.739	0.00617

### Egg density evaluation

**Figure 5:**
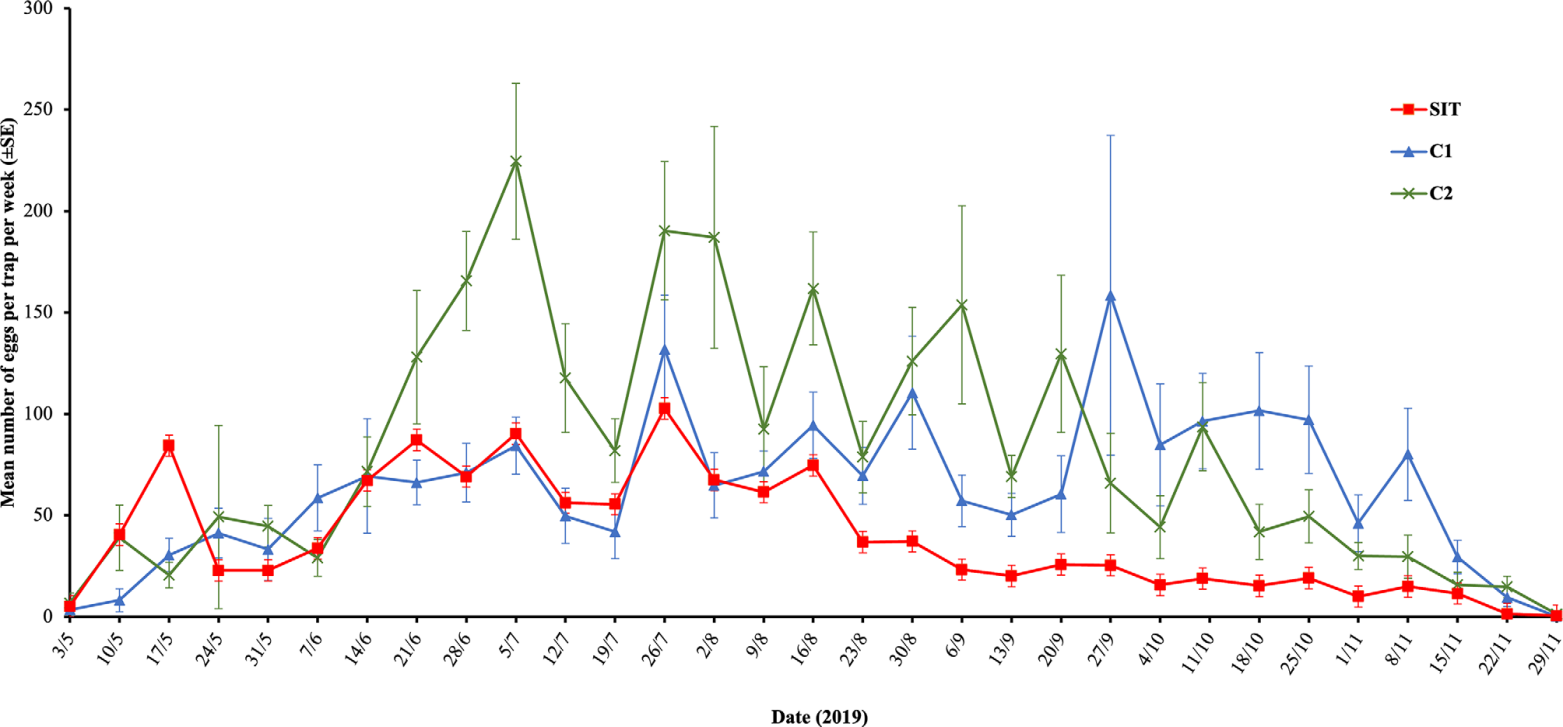
The seasonal trend of egg density (mean ± SE) collected at SIT and control plots C1 and C2.

The average egg density recorded in the SIT plot was similar to that of control 2 (Table 5, *p* = 0.82) but initially higher than in control 1 (*p* < 0.01) at the beginning of the trial. Egg density increased over time in controls 2 and 1 (*p* < 0.01), whereas it decreased in the SIT plot (*p* < 0.01), demonstrating significant suppression (Fig. 5). During the period from mid-June to early September, the egg reduction rate exhibited a significant increase, reaching 78%, and this elevated rate persisted within the range of 70–78% for up to 4 weeks following conclusion of the releases (Fig. 4).

**Table 5:** Fixed-effects coefficients of a mixed-effect Poisson model of the impact of treatment, time and their interaction on the density of eggs of *Aedes albopictus* (1,426 observations, 60 traps, 24 collection dates, Control 2 site used as a reference).

Fixed effects	Value	SE	*z*-value	*p*-value
Intercept	3.547625	0.340171	10.429	<2e−16
Control 1	−0.859416	0.278708	−3.084	0.00205
SIT	0.054842	0.241428	0.227	0.82030
Time	0.240769	0.079693	3.021	0.00252
Control 1: Time	0.128733	0.006426	20.034	<2e−16
SIT: Time	−0.315907	0.005966	−52.952	<2e−16

### Oviposition positivity index (OPI)

**Figure 6:**
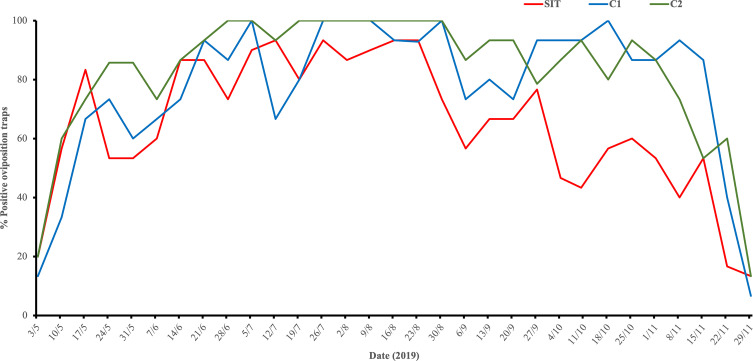
Weekly distribution of oviposition positivity index (OPI) at the release (SIT) and control plots C1 and C2 from 03 May to 29 November (2019).

The percentage of OPI varied at similar rates from May until mid-August among the three plots, but declined substantially from this point to the end of the season in the SIT compared to two control plots (Fig. 6). Also, it is noteworthy that the POI in the SIT plot did not reach 100% positivity at any point during the trial.

## Discussion

In 2018, toward the end of the mosquito season, a door-to-door strategy was implemented in Vravrona (target area) to reduce breeding sites in both private and public areas before the application of the pilot release of sterile males [2, 34]. The results from the suppression trial in 2019 highlighted the efficacy of the SIT, as part of an AW based approach, even in a small plot and in a one-year trial. The integration of SIT with complementary interventions has demonstrated the potential for achieving a substantial reduction in *Ae. albopictus* populations. This approach not only mitigates mosquito nuisance, but it also holds promise in significantly diminishing the incidence and transmission of vector-borne diseases [5, 34, 37]. Several countries worldwide have undertaken pilot projects and field trials involving SIT to reduce populations of *Ae. albopictus* using local production facilities for the sterile males such as Italy [9], Spain [35], Mexico [11] and China [38].

Following our previous results in 2018, all sterile males released in 2019 were produced at the CAA (Italy) facility and transported to the release plot [29]. In the current study, for the first time, a successful suppression trial was applied using sterile males from a local Greek strain (Fig. 1) that was mass reared in a facility located in another European country (Italy). Our previous studies indicated the significant challenges associated with transporting sterile males for durations exceeding 24 h. Such extended transportation periods often lead to high mortality rates and increased stress among the sterile males involved, but in our case study, we have significantly reduced the packaging, transportation duration, and release processes to approximately 21 h to minimise the risks of mortality and stress of sterile males. By shortening the transportation time, while safeguarding the quality of the sterile males, we can confirm that we made significant progress towards improving the efficiency and efficacy of our operations. Through planning and implementation, we have successfully established a long-distance transportation method for sterile males, which could also be applied to other European and Mediterranean countries [5].

A progressive increase in efficacy of SIT measured as percentage of induced egg sterility and egg reduction in comparison to control was observed, which lasted for at least 3 weeks after the cessation of the releases. This suggests that the SIT approach can effectively reduce mosquito populations over time to overcome possible compensation effects. During the first 7 weeks of the trial, no reduction in the local *Ae. albopictus* population was observed. Additional factors, including reduced larval density and potential migration, could influence the observed population dynamics. Nevertheless, since the SIT plot is a relatively isolated area, we assume that this increase was probably related to compensation, a biological mechanism that allows populations to offset losses through increased reproduction and survival. The effect of compensation could have reduced the impact of the first releases, resulting in an apparent lack of population reduction, as observed recently in four other countries [12].

In this study, both induced sterility and egg reduction rates reached a maximum of 78%. In a previous 10-week trial conducted in the same plot (weeks 37–47 of the year 2018, in a smaller release plot of 5 ha), induced sterility exhibited fluctuations within the range of 40–84% without displaying a clear decreasing trend, and it did not result in any significant reduction of egg density [2]. This is in line with previous studies conducted in Italy, which demonstrated that an induced egg sterility below 60% did not lead to a reduction in the egg density [8]. This is also in line with observations from increased larval mortality in *Ae. albopictus* and is considered to be related to density-dependent compensation of mortality [14, 21, 30]. Specifically, a reduction in egg sterility could lead to a decrease in larval density within breeding sites, which can have varying effects on the overall adult population density.

In the phased conditional approach developed by the International Atomic Energy Agency (IAEA) for SIT suppression trials against *Aedes* mosquitoes, it is recommended that the number of sterile males released per week exceeds the requirements determined based on the selected field plot and defined release frequency [13]. In light of our results, these numbers should be calculated with the aim of achieving a minimum of 60% sterility.

Wild females immigrating from the surrounding plots is another important factor that could affect the efficacy of SIT field suppression trials, particularly when the size of the study plots is small as it is in the present case [17, 22]. Controlling this parameter is not easy, even though it is crucial for selecting pilot release sites [34]. In the 2018 trials, we targeted only half of the current release plot that is isolated from other villages, with a lower impact on egg hatch and without any reduction of mosquito densities [2]. Extending the release to the full plot clearly decreased the propensity of females immigrating from the surrounding environment and led to much stronger impact on the target population. The ovitraps are the most cost-effective monitoring tools and allow the evaluation of both the density of the wild population and induced sterility [33].

To achieve successful egg hatching outcomes, it is imperative to emphasise the crucial steps of egg collection and oviposition substrate storage. During our recent project in Chania, situated on the island of Crete (Greece), we collected eggs that were subsequently transported and hatched at the Benaki Phytopathological Institute in Athens (Greece) [7]. By prioritising these essential procedures, we were able to optimise our egg hatching process and achieve favourable results. Meticulous attention to detail during egg collection ensures the acquisition of healthy and viable eggs, significantly contributing to the success of subsequent hatching efforts (avoiding issues such as egg desiccation or damage). Furthermore, the proper storage of the oviposition substrate plays a critical role in maintaining the necessary conditions for optimal embryo development.

Moreover, the number of eggs collected can effectively predict the wild population density and therefore provide an estimation of the sterile to wild ratio in the field [6]. Another important aspect to evaluate in an SIT project is the quality of the released males, which determines their mating competitiveness. The evaluation must take into account the lag between releases and their observed effect (at least 3 weeks due to the time needed by the newly emerged females to replace the old already mated females) and, in non-isolated plots, the immigration of wild fertile females from the surrounding plots [13, 17]. In future control efforts in Greece, adult traps will be deployed to assess this parameter, following a procedure that is currently used in the Attica region with great success to monitor the population dynamics of wild *Ae. albopictus* [2].

In conclusion, the SIT is a method that involves releasing large numbers of sterile males to outcompete wild males in mating with females. Over time, this leads to a decline in pest populations, and in some cases, their complete eradication [18, 36]. The SIT has been widely used to suppress or eradicate populations of major insect pests in agriculture, livestock, and human health, with notable success. It is important to mention that there is no standardised protocol for the release of sterile mosquitoes, and the methodology employed in this study can be considered a prototype. Like any innovative technique, continuous improvements and adjustments are being made to optimise its application, including aspects such as the transportation of sterile males and the assessment of egg hatch rates. In our current study, we released sterile males on a weekly basis for 22 weeks, based on long distance production of sterile males (transported sterile males) resulting in an egg reduction even after the end of the releases.

A limitation of our study was the lack of calculations for the sterile:wild-type male ratio. This limitation arose from our inability to implement Mark Release Recapture (MRR) trials due to limited human resources. Despite this lack of information, we demonstrated that sterile male mosquito releases (SIT implementation) reduced both egg hatching rates and the target mosquito population.

The results obtained from this study provide valuable insights for future SIT programmes targeting *Ae. albopictus*. Additionally, the adopted methodology can serve as a foundational framework for refining and enhancing release protocols. The consideration of sterile male mosquito transportation is crucial to this improvement. However, the shift from a small-scale trial to a large-scale programme poses new challenges in the associated logistics, such as shipment costs that need to be prioritised and investigated. Our perspective is to establish local production of sterile males, progressively expanding pilot trials, and assessing the cost-effectiveness of integrating SIT into the national integrated vector control strategy.

## Supplementary materials

The Supplementary materials of this article are available at https://www.parasite-journal.org/10.1051/parasite/2024020/olm. *Video 1*: Release of *Aedes albopictus* sterile males in the field (Credit: George Balatsos).
